# Remote BV Management via Metagenomic Vaginal Microbiome Testing and Telemedicine

**DOI:** 10.3390/microorganisms13071623

**Published:** 2025-07-09

**Authors:** Krystal Thomas-White, Genevieve Olmschenk, David Lyttle, Rob Markowitz, Pita Navarro, Kate McLean

**Affiliations:** Evvy, New York, NY 10001, USA

**Keywords:** bacterial vaginosis, telemedicine, vaginitis, metagenomics, diagnostics

## Abstract

Bacterial vaginosis (BV) affects 30% of women annually, but many face barriers to in-person care. Here we present real-world outcomes of remote BV diagnosis and management through self-collected vaginal microbiome (VMB) testing and telemedicine visits, focusing on symptom resolution, recurrence, and overall microbial shifts. Among the 1159 study participants, 75.5% experienced symptom resolution at four weeks when managed with our algorithm-guided treatment protocol. At a median follow-up of 4.4 months after the initial visit, 30.0% of patients experienced recurrent BV, which is lower than the typical recurrence rates seen in historical in-person cohorts. Across the entire cohort, metagenomic data demonstrated a significant increase in *Lactobacillus* abundance (mean of 32.9% to 48.4%, *p* < 0.0001) and a corresponding decrease in BV-associated taxa such as *Gardnerella*, *Prevotella*, and *Fannyhessea*. A PERMANOVA of pairwise Bray–Curtis distances showed significant separation between pre-and post-treatment samples (pseudo-F = 37.6, *p* < 0.0001), driven by an increase in *Lactobacillus*-dominated samples. Treatment adherence was high (a total of 78% reported perfect or near-perfect adherence), and adverse events were generally mild (in total, 22% reported vaginal irritation, and 13% reported abnormal discharge). These results demonstrate that Evvy’s at-home metagenomic platform, paired with telemedicine and a smart treatment algorithm, delivers robust clinical and microbial outcomes. This work offers a novel approach to managing bacterial vaginosis, a challenging condition characterized by persistently high recurrence rates.

## 1. Introduction

Bacterial vaginosis (BV) represents the most prevalent vaginal infection, affecting approximately 30% of individuals with vaginas each year [1]. Clinically, BV is characterized by vaginal irritation and excessive malodorous discharge [2]. It can also have a significant psychosocial impact, with patients reporting a substantially diminished quality of life, higher levels of depression and anxiety, as well as profound interpersonal and social distress [3]. Beyond its immediate symptomatology, BV exemplifies a complex microbiological dysbiosis associated with long-term clinical ramifications, such as increased susceptibility to sexually transmitted infections [4,5], impacts on fertility [6,7,8], increased risk of adverse pregnancy outcomes [9,10,11], and cervical cancer progression [12,13]. Access to in-person gynecologic care can be limited by provider shortages, geographic care deserts, or patient reluctance, leaving many women untreated or forced into empirical trials of multiple courses of antibiotics. Telemedicine, paired with self-collected at-home microbiome sampling, offers a practical solution to these barriers.

Standard treatment for BV includes either topical or oral antibiotic treatment with metronidazole or clindamycin. These treatments result in a 70–85% response rate within 1 month [14]. However, recurrence rates are notably high, with 45% recurring within 3 months [15] and over 50% recurring within 6 months [14]. The persistent cyclical nature of treatment and recurrence underscores the therapeutic limitations of current interventional strategies, potentially reflecting an incomplete understanding of the complex microbiological dynamics underlying vaginal dysbiosis.

The vaginal microbiome (VMB) comprises a complex community of bacteria and fungi colonizing the vaginal environment. These microorganisms function as a critical protective barrier by inhibiting pathogens and maintaining homeostasis [16,17]. An optimal VMB is canonically characterized by a predominance of *Lactobacillus* species (primarily *L. crispatus*, *L. jensenii*, *L. gasseri*, *L. paragasseri*, and *L. mulieris*) [17]. In the absence of protective lactobacilli, a diverse anaerobic community emerges which can include *Gardnerella* species (*G. vaginalis*, *G. leopoldii*, *G. piotii*, and *G. swidsinskii*), *Prevotella*, *Fannyhessea* (previously *Atopobium*), *Mobiluncus*, *Megasphaera*, *Finegoldia*, *Fusobacterium*, *Mycoplasma*, *Ureaplasma*, *Porphyromonas*, *Sneathia*, and others [18,19]. There is ongoing research to determine the impact of *L. iners* on the development of BV.

Due to the polymicrobial nature of BV, no single organism has been conclusively identified as the primary etiological agent [20]. Instead, various microbiome profiles can manifest similar symptomatic presentations, rendering accurate diagnosis and treatment challenging. In traditional clinical settings, even with careful physical examination, microscopy, and PCR-based testing, the rate of misdiagnosis for BV has been reported to be well over 50% [21].

In this study, we present a novel diagnostic and therapeutic approach to addressing BV via telehealth. We hypothesize that the metagenomic sequencing of self-collected vaginal samples, delivered via telemedicine, can reliably diagnose BV and inform remote antibiotic/adjunctive treatment. We further posit that home-driven microbiome analysis will demonstrate overall microbial restoration (an increase in *Lactobacillus* and a decrease in BV-associated taxa) following remote therapy, thus validating a fully remote, medically sound BV care pathway.

## 2. Materials and Methods

### 2.1. Patient Selection

All study participants provided informed consent, and study procedures were conducted in accordance with protocols approved by a federally accredited Institutional Review Board (IRB# 20220118.evvy).

Study inclusion required patients to have undergone vaginal microbiome characterization via shotgun metagenomic sequencing and received a diagnosis of BV between November 2022 and July 2024. The BV diagnosis was established based on presenting symptomatology aligned with the established literature, primarily malodorous vaginal discharge, in conjunction with metagenomic evidence of moderate relative abundance of BV-associated organisms, such as *Gardnerella* or *Prevotella* [19]. Eligible patients initiated treatment through Evvy’s telemedicine platform, receiving either metronidazole or clindamycin along with other personalized treatments, and completed follow-up metagenomic testing within one year of initial treatment. The selection of the antibiotic type was dependent on a constellation of factors, including patient-reported allergies and preferences, the relative abundance of specific bacterial species, and any co-occurring microbes known to be more susceptible to one antibiotic over the other.

Due to telemedicine practice limitations and safety considerations, patients with diabetes, with a current cancer diagnosis, who were breastfeeding, presenting HIV, having an immunocompromised status, experiencing pregnancy, presenting untreated sexually transmitted infections, or with a recent positive STI test were excluded from the platform and directed to seek in-person clinical care. Patients with test results showing evidence of an active fungal infection were assigned different treatment protocols that included treatment with anti-fungals. These populations were consequently not represented in the study cohort.

### 2.2. Description of Clinical Care Protocols

Patients presenting with vaginal symptoms and a dysbiotic vaginal microbiome are eligible for clinical care through Evvy’s telehealth platform. The platform integrates comprehensive metagenomic data analysis with patient-reported medical history to generate evidence-based, precision treatment protocols. While the platform serves patients with various forms of vaginitis (including vulvovaginal candidiasis, aerobic vaginitis, and genitourinary syndrome of menopause), this study specifically focuses on BV treatment outcomes.

Given our symptomatic patient population, BV prevalence is notably high. Treatment protocols are individually tailored, comprising both primary antibiotic therapy and targeted adjunctive interventions. As noted above, antibiotic selection is guided by dual objectives: the suppression of pathogenic microorganisms while preserving existing beneficial *Lactobacillus* communities. Evidence-based adjunctive treatments, such as boric acid, vaginal estrogen, probiotics, and prophylactic anti-fungals, are included based on the patient’s history, symptoms, and microbiome profile. All prescriptions are prescribed at the discretion of a licensed clinician, are made with medical-grade compounds, and are dispensed through an accredited pharmacy (Precision Compounding, New York, NY, USA).

Research indicates that patients with recurrent vaginitis experience significant psychosocial isolation, potentially compromising treatment outcomes [22]. The platform addresses this through comprehensive patient support, including evidence-based educational resources, symptom monitoring, and individualized health coaching sessions.

### 2.3. Measuring Observed Response and Recurrence Rate—Treatment Response Evaluation

Treatment response was assessed through patient-reported post-treatment questionnaires using a 5-point Likert scale [23]. Patients were asked, “Since the start of treatment how have your vaginal symptoms changed?” on a scale of 1 (significantly better) to 5 (significantly worse). Patients were classified as responders, if they reported significant or moderate improvement, or non-responders, if they reported unchanged or worsened symptoms. Response data were collected on average 4.4 months post-initial treatment during post-treatment assessment.

### 2.4. Symptom Severity Assessment

Symptom severity was quantitatively evaluated at each sampling timepoint by using a 14-parameter symptom questionnaire that included the following symptoms: excessive discharge, odorous discharge, vaginal pain, vulvar pain, vulvar erythema, vaginal edema, external and internal itchiness, vaginal dryness, vaginal burning, vulvar burning, dyspareunia, and dysuria. Patients rated each symptom on a scale of 0–3 (0 = absent, 1 = mild, 2 = moderate, and 3 = severe). These ratings were aggregated to generate a comprehensive symptom score. Therapeutic efficacy was determined by calculating the absolute change in symptom scores between pre- and post-treatment testing.

### 2.5. Recurrence Monitoring

Recurrence data were collected through multiple channels: direct platform re-engagement, structured follow-up questionnaires, and a systematic review of clinical documentation. Recurrence on Evvy’s platform was defined as the subsequent prescription of metronidazole or clindamycin for vaginitis. Additionally, 193 post-care clinical notes were systematically reviewed for the documentation of external antibiotic prescriptions or provider consultations. Finally, a supplementary email-based follow-up survey captured whether patients sought treatment outside our platform.

### 2.6. Treatment Adherence Measurement

Treatment adherence was evaluated using a patient-reported 5-point scale, where 5 represented strict protocol adherence and 1 indicated significant protocol deviation.

### 2.7. Sample Collection and Sequencing Methodology

Vaginal specimens were self-collected using standardized collection kits (Copan, Murrieta, CA, USA) following previously established protocols [24]. Samples were processed, which included chemical and mechanical lysis, host depletion, and DNA extraction using an automated extraction handling instrument. NGS libraries were prepared, multiplexed, quality-checked, and sequenced on the Illumina NovaSeq 600 (Illumina, San Diego, CA, USA). Shotgun metagenomic sequencing was performed using a CLIA/CAP/CLEP-certified analytical pipeline (Microgen DX, Lubbock, TX, USA). Patient data collection included questionnaires documenting symptomatology, relevant clinical diagnoses, demographic information, and treatment response metrics at post-treatment assessment.

### 2.8. Bioinformatic Analysis

Taxonomic relative abundance profiles were generated via a custom bioinformatic pipeline [24]. Raw sequencing reads were pre-processed through quality filtering and adapter trimming steps, followed by a host depletion step which filtered out human sequences by aligning them to a human reference genome (GRCh38) [25]. The cleaned and filtered reads were then used to generate genus- and species-level taxonomic abundance profiles by aligning reads to a proprietary reference database generated from a large, curated collection of vaginal microbial genomes [24]. The resulting abundance tables were filtered at a minimum relative abundance cutoff of 0.75% prior to downstream analysis.

### 2.9. Statistical Analysis

Genus-level taxonomic analyses were performed by calculating combined relative abundances of key bacterial genera (*Gardnerella*, *Lactobacillus*, *Prevotella*, and *Fannyhessea*) and broader biological groups (aerobic and anaerobic bacteria). Species-level analyses were conducted for individual *Gardnerella* and *Lactobacillus* species. Overall shifts in taxon abundances (pre- vs. post-treatment) were evaluated using Wilcoxon signed-rank tests on paired samples. *Lactobacillus* genus abundance, BV-associated taxa (*Gardnerella*, *Prevotella*, *Fannyhessea*), and *Gardnerella* species were assessed across the entire cohort. Distribution patterns of combined taxa and individual species were visualized using enhanced box plots (boxen plots). Mean values are reported in the main text, with corresponding medians and interquartile ranges (IQRs) provided in Supplementary Table S3. Significance for paired changes (pre- vs. post-treatment) was defined as *p* < 0.05.

Overall community structure changes (pre- vs. post-treatment) were visualized using UMAP, and a PERMANOVA test on pairwise Bray–Curtis distances was used to assess separation between pre- and post-treatment samples [26,27]. Demographic and clinical variables were analyzed using R (version 4.4.2) and Python (version 3.12). Because this was a retrospective observational study, no power analysis was performed [28].

## 3. Results

### 3.1. Treatment Response Analysis

This retrospective observational study included 1159 patients, of whom 75.5% (N = 875) reported significant or moderate improvement and 24.5% (N = 284) reported no change or a worsening in symptoms. Antibiotic selection comprised either metronidazole (metro) (N = 535) or clindamycin (clinda) (N = 624).

### 3.2. Demographics

The participants in the study were distributed across all major geographic regions of the United States, including the Northeast, South, Midwest, and West. The majority of participants were White, with a mean age of 38 and a BMI of 25. Additional demographic characteristics—including menopausal status, pregnancy status, number of sexual partners, contraceptive use, comorbidity profiles, and use of vaginal products in the 30 days prior to treatment—are summarized in Table 1 and Supplementary Tables S1 and S2.

### 3.3. Clinical Symptom Resolution

**Figure 1:**
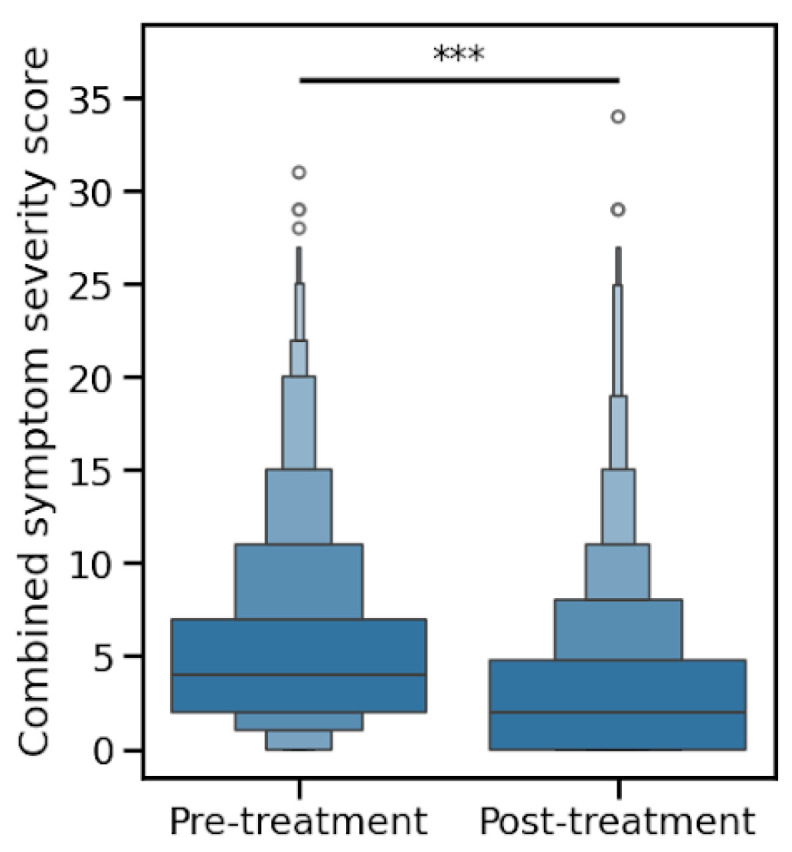
Combined symptom severity scores before and after treatment. Symptom severity scores significantly decreased following treatment, demonstrating overall symptom improvement across the cohort. Complete statistical analyses are provided in Supplementary Table S3. Statistical significance: *** *p* < 0.0001 (Wilcoxon rank test).

Patients were asked to rate 12 urogenital symptoms on a scale of 0-3 (not experiencing, mild, moderate, or severe). The mean summed symptom scores decreased after treatment, from 5.5 to 3.4 (*p* < 0.0001), as shown in Figure 1.

### 3.4. Recurrence Rate Analysis

The average interval between care and post-treatment testing was 4.4 months. For the recurrence diagnosed on our platform, 30.0% (348/1159) recurred at the time of their repeat test. This recurrence rate was observed across the following time periods: <3 months (29.6%), 3–4 months (30.8%), and 4–5 months (28.6%) post-treatment initiation (Table 2). A notably lower recurrence rate (22.6%) was documented in patients evaluated between 5 and 6 months. This corresponds with the previous literature demonstrating peak recurrence risk within 3–6 months post-treatment [14]. Of the 811 remaining patients with no known recurrence on our platform, all tested negative for BV at the time of their post-treatment test.

Ten individuals received treatment for BV through Evvy’s platform between their follow-up test and up to 1 year after their original diagnosis. We collected information on 27 patients that received care from an outside provider for BV anytime after their initial BV diagnosis on our platform. In total we have documented evidence that 33.2% (385/1159) of patients had BV recurrence up to 1 year following the initial diagnosis on our platform.

While the follow-up information for the 811 with negative repeat testing and no known recurrence for a full 12 months is not complete, it, nevertheless, suggests a lower recurrence rate than typically reported.

### 3.5. Overall Vaginal Microbiome Shifts Pre- vs. Post-Treatment

Across all 1159 paired baseline and follow-up samples, significant changes in community composition were observed after remote, algorithm-driven BV therapy. These shifts reflect both a reduction in dysbiotic taxa and an enrichment in protective *Lactobacillus* species.

### 3.6. Alpha Diversity

**Figure 2:**
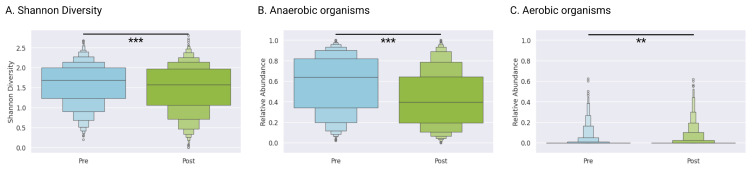
Overall microbial community changes pre- vs. post-treatment. (**A**) Shannon diversity indices, (**B**) pathogenic anaerobe abundance, and (**C**) pathogenic aerobe abundance at baseline and follow-up assessment. Organisms classified as aerobic or anaerobic are detailed in Supplementary Table S4. Complete statistical analyses are provided in Supplementary Table S3. Statistical significance: ** *p* < 0.001, and *** *p* < 0.0001 (Wilcoxon rank test).

The Shannon diversity index decreased from a mean of 1.59 (IQR 1.23–2.00) at baseline to 1.49 (IQR 1.07–1.97) post-treatment (*p* < 0.0001, Wilcoxon signed-rank test), indicating overall community simplification following antibiotic and adjunctive interventions (Figure 2A).

### 3.7. Anaerobic and Aerobic Taxa

Anaerobic genera (e.g., *Gardnerella* and *Prevotella*) decreased significantly from a mean of 57.8% (IQR 34.2–82.0) pre-treatment to a mean of 43.1% (IQR 19.6–64.6) post-treatment (Figure 2B). Aerobic genera (e.g., *Staphylococcus* and *Streptococcus*) remained low with mean abundances of 1.4% (IQR 0–0) before treatment and 2.0% (IQR 0–0) after treatment. Though we saw statistically significant changes in the abundance of these taxa overall (*p* = 0.0005), the effect was quite small, confirming that therapy primarily targeted anaerobic dysbiosis rather than broadly altering the aerobic community (Figure 2C).

### 3.8. Lactobacillus Genus

**Figure 3:**
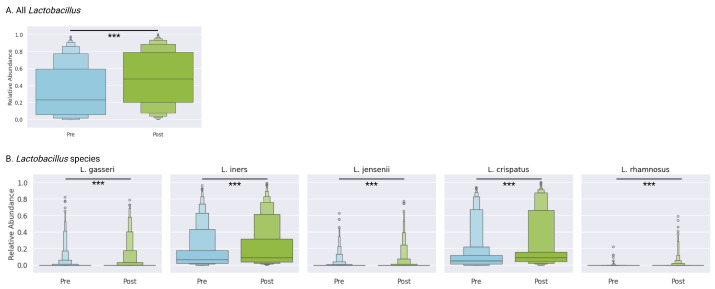
Overall *Lactobacillus* genus and species shifts pre- vs. post-treatment. (**A**) Total *Lactobacillus* genus abundance and (**B**) species-specific abundance of vaginal-associated *Lactobacillus* taxa at baseline and follow-up assessment. Complete statistical analyses are provided in Supplementary Table S3. Statistical significance: *** *p* < 0.0001 (Wilcoxon rank test).

The mean relative abundance of *Lactobacillus* increased significantly from 32.9% (IQR 5.8–59.6) at baseline to 48.4% (IQR 20.4–79.1) post-treatment (*p* < 0.0001), reflecting the restoration of protective flora (Figure 3A). This shift toward *Lactobacillus* dominance suggests successful ecological recovery following targeted antibiotic therapy and adjunctive support.

### 3.9. Lactobacillus Species-Level Dynamics

At the species level, several *Lactobacillus* taxa demonstrated significant increases: *L. crispatus* increased from 12.6% (IQR 1.3–12.0) to 20.2% (IQR 4.2–15.6) (*p* < 0.0001), and *L. iners* increased from 15.8% (IQR 2.3–17.7) to 21.2% (IQR 3.7–31.4) (*p* < 0.0001). A subset of *Lactobacillus* species, *L. jensenii*, *L. gasseri*, and *L. rhamnosus*, were less prevalent, and were all present in less than 25% of samples both before and after treatment, though in all cases, there were small but statistically significant increases (*p* < 0.0001) (Figure 3B). These coordinated gains underscore the reestablishment of *Lactobacillus* species.

### 3.10. BV-Associated Taxa

**Figure 4:**
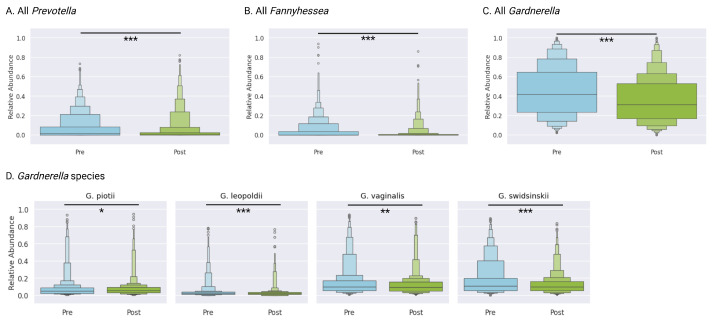
Overall shifts in BV-associated taxa (*Gardnerella*, *Prevotella*, and *Fannyhessea*) pre- vs. post-treatment. Comparative abundance of (**A**) *Prevotella*, (**B**) *Fannyhessea*, and (**C**) *Gardnerella* genera at baseline and follow-up baseline, with (**D**) species-level analysis of *Gardnerella* taxa. Complete statistical analyses are provided in Supplementary Table S3. Statistical significance: * *p* < 0.05, ** *p* < 0.001, and *** *p* < 0.0001 (Wilcoxon rank test).

The aggregate relative abundance of key BV-associated taxa declined markedly. *Gardnerella* spp. decreased from a mean of 44.8% (IQR 23.8–64.8) at baseline to 35.7% (IQR 16.9–53.1) post-treatment (*p* < 0.0001). *Prevotella* dropped from 7.1% (IQR 0–8.4) to 4.6% (IQR 0–2.7) (*p* < 0.0001). *Fannyhessea* (previously *Atopobium*) was less prevalent, with mean abundances of 4.3% before treatment and 1.8% after treatment, though there was a small but statistically significant decrease (Figure 4).

### 3.11. Gardnerella Species

Overall *Gardnerella* abundance decreased from 44.8% to 35.7% (*p* < 0.0001). At the species level, *G. swidsinskii* declined from 17.5% (IQR 5.5–20.3) to 12.7% (IQR 5.4–16.4) (*p* < 0.0001), *G. leopoldii* decreased from 4.4% (IQR 1.3–4.0) to 3.1% (IQR 1.2–3.5) (*p* < 0.0001), and *G. vaginalis* declined from 14.9% (IQR 5.4–17.1) to 12.2% (IQR 5.1–15.6) (*p* = 0.0005). Trends for *G. piotii* (8.1% to 7.7%, *p* = 0.05) showed non-significant reductions (Figure 4D). Collectively, these reductions contributed to the overall decline in BV-associated burden.

### 3.12. Community Structure (Beta Diversity)

The PERMANOVA of pairwise Bray–Curtis distances revealed a significant global shift from baseline after treatment (pseudo-F = 37.6, *p* <= 0.001, PERMANOVA with 999 permutations). Uniform Manifold Approximation and Projection (UMAP) was used to visualize the overall community structure and the clustering of samples with similar compositions. Post-treatment samples tended to belong to more *Lactobacillus*-dominant clusters, whereas clusters corresponding to dominance by BV-associated taxa were predominantly composed of pre-treatment samples, consistent with a community reconfiguration toward health (Supplementary Figure S1).

### 3.13. Treatment Adherence and Adverse Events

**Figure 5:**
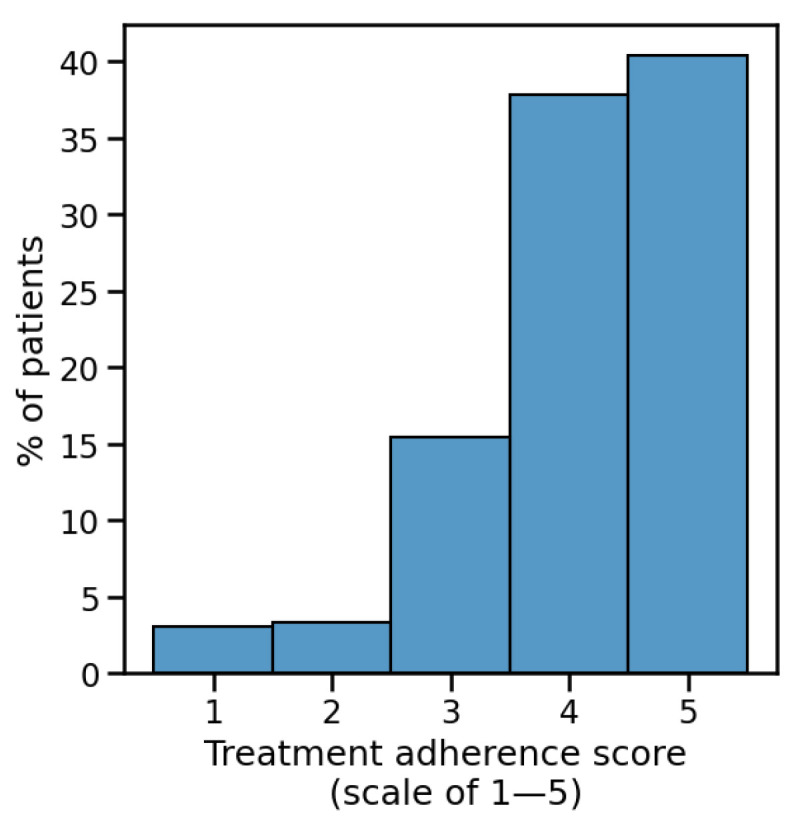
Rates of adherence. The distribution of self-reported treatment adherence scores, showing that the majority of patients were either totally compliant (5) or highly compliant (4).

Adverse events were generally mild (Table 3) and did not correlate with treatment choice. Overall, 56% of patients reported zero side effects, 30% reported one side effect, and 14% reported multiple side effects. Most common events were vaginal irritation (22%) and abnormal discharge (13%). Treatment adherence was high: a total of 78% of patients reported strict adherence (scores of 4–5; Figure 5).

## 4. Discussion

This large-scale analysis of real-world data provides compelling evidence for telehealth-driven, microbiome-informed approaches to BV management. This platform’s integration of high-resolution metagenomic sequencing with tailored therapeutic protocols achieved a 75.5% response rate, comparable to or exceeding traditional care outcomes [14]. Notably, our 4.4-month (average) follow-up exceeds the typical 1-month window used in clinic-based studies [14], suggesting sustained benefit. The sustained treatment response suggests that this telehealth platform’s treatment response may be even more robust than in-person published protocols. All patients exhibited improvement in vaginal community structure, with increased *Lactobacillus* and decreased BV-associated taxa. Similarly, the observed 30.0% recurrence rate (at an average of 4.4 months) represents an improvement over historical rates of 45–50% for in-person care reported in the literature that were measured within shorter follow-up windows [14,15]. Our findings demonstrate the viability of telehealth approaches for the achievement of sustained clinical response.

High adherence with Evvy’s telemedicine-guided treatment protocol, which was common despite the remote nature of the care delivered, was associated with better clinical and microbial outcomes. This highlights the critical role of patient engagement in sustaining vaginal health via remote approaches. Adverse events were generally mild—most commonly vaginal irritation or discharge—and occurred at similar frequencies regardless of antibiotic choice.

As the largest real-world evaluation of remote, microbiome-informed BV management to date, this study demonstrates that high-resolution, at-home sequencing paired with targeted treatment algorithms enables clinicians to deliver personalized, evidence-based care in the absence of in-person evaluation. By leveraging shotgun metagenomic data and systematic bioinformatic analyses, providers can address both immediate symptoms and minimize recurrence risk through the strategic modulation of the vaginal microbiome. With recurrent BV accounting for significant downstream costs (including repeated clinic visits, fragmented care, and psychosocial distress), a telehealth-enabled model can mitigate these burdens. By enabling at-home sampling and remote provider interaction, we eliminate travel/time costs and reduce barriers—especially in rural or underserved communities.

### 4.1. Limitations

Several limitations of this real-world evidence study warrant discussion. The observational nature of the study design introduced variability in follow-up windows, preventing the standardization of sampling timepoints. Follow-up data were available for only 33.2% of patients, potentially introducing bias in recurrence rate estimates. No validated questionnaires exist to assess BV symptoms, and while standard questionnaire formats were used in this study [23], future research would benefit from a validated BV questionnaire. The telemedicine-based approach, while enabling broad accessibility, limited direct clinical assessment. Additionally, concurrent treatment from external providers could not be definitively excluded.

Despite these limitations, these findings have important implications for clinical practice, suggesting that the optimization of BV treatment outcomes may be achieved through microbiome-guided, remote therapeutic strategies. As our understanding of the vaginal microbiome’s role in health and disease continues to expand, the integration of comprehensive diagnostics with personalized treatment protocols may enhance the standard of care in managing vaginal dysbiosis.

### 4.2. Future Directions

This comprehensive analysis of microbiome-guided BV treatment establishes a foundation for several critical areas of future investigation.

First and foremost, prospective studies comparing the outcomes of metagenomics guided telehealth-based BV management against traditional in-person care are crucial. These studies should assess not only recurrence rates but also factors such as patient satisfaction, cost-effectiveness, and access to care in underserved or rural communities. By demonstrating that telemedicine offers equivalent or superior outcomes compared to conventional methods, we can support the broader adoption of remote care pathways, ultimately improving access and reducing healthcare disparities for individuals with BV.

The long-term monitoring of the vaginal microbiome is another critical area for future investigation. Extended follow-up beyond the typical 4–6 months post-treatment will provide insights into the durability of microbial restoration achieved via telehealth. By incorporating regular at-home sampling and real-time analytics, we can identify early shifts toward dysbiosis, enabling timely interventions before recurrence occurs.

Additionally, addressing BV recurrence may benefit from integrating partner treatment protocols [29]. Evidence suggests that untreated partners can contribute to reinoculation, thus increasing recurrence rates [14]. Future studies should explore whether incorporating partner screening and coordinated treatment further reduces recurrence. The convenience of at-home sampling for both partners could streamline dyadic interventions, enhancing long-term microbiome stability and potentially lowering recurrence rates.

As research advances, we should also examine behavioral and environmental triggers that may disrupt the vaginal microbiome. By tracking lifestyle factors such as stress, sexual activity, and antibiotic use alongside serial microbiome profiles, future studies could reveal modifiable factors contributing to BV recurrence. This information would allow clinicians to refine personalized maintenance strategies beyond initial antimicrobial therapy.

Lastly, longitudinal studies are essential to evaluating whether microbiome-guided, telehealth approaches confer protection against adverse reproductive health outcomes. Specifically, the investigation of the relationship between restored vaginal microbiome composition and risks of preterm birth, assisted reproductive technology outcomes, and sexually transmitted infection acquisition would establish the broader clinical impact of this therapeutic strategy.

## 5. Conclusions

Evvy’s integration of at-home metagenomic sequencing, telemedicine, and algorithm-driven therapy delivers high symptom resolution, microbiome restoration, and low recurrence, laying the groundwork for a new standard in BV management. Ongoing trials will assess direct head-to-head comparisons with in-clinic care and long-term patient adherence.

## Figures and Tables

**Table 1:** Demographics.

	Total (N = 1159)
Age (ave)	38.7
BMI (ave)	25.3
Gender Identity	
Woman	99.4% (1152)
Non-binary	0.5% (6)
Prefer not to say	0.1% (1)
Race/Ethnicity	
White	78.3% (907)
Hispanic/Latino	12.7% (147)
Black or African American	9.2% (107)
Asian	4.3% (50)
Middle Eastern	1.5% (17)
American Indian or Alaskan Native	1.4% (16)
South Asian	0.7% (8)
Southeast Asian	0.6% (7)
Native Hawaiian or Other Pacific	0.4% (5)
Islander	
Prefer not to say	1.8% (21)
Other	0.9% (10)
Menopause Status	
Premenopausal	78.4% (909)
Menopausal	8.6% (100)
Perimenopausal	10.5% (122)
Other menopausal statuses ^1^	2.4% (28)

^1^ This includes individuals who had a hysterectomy, uterine ablation, primary ovarian insufficiency, and chemotherapy.

**Table 2:** Temporal analysis of BV recurrence following initial treatment.

Testing Time Frame	Recurred—BV Diagnosed on Follow-Up Test *	Did Not Recur—No BV Diagnosis on Follow-Up Test *	Recurrence Rate	Total
Overall	348	811	30.0%	1159
<3 months (<12 weeks)	40	95	29.6%	135
3–4 months (12–16 weeks)	152	342	30.8%	494
4–5 months (16–20 weeks)	73	182	28.6%	255
5–6 months (20–24 weeks)	24	82	22.6%	106
>6 months (>24 weeks)	59	110	34.9%	169

* Time-stratified recurrence rates of bacterial vaginosis, defined by subsequent antimicrobial prescription (metronidazole or clindamycin) at follow-up evaluation. Data reflect care episodes within our telemedicine platform. Follow-up intervals are calculated from initial treatment initiation to subsequent assessment. Numbers at each timepoint are not cumulative.

**Table 3:** Patient-reported adverse events. Specific symptoms are listed in Supplementary Table S5.

	No Side Effects	Only 1 Side Effect	More than 1 Side Effect
Total	57.5% (666/1159)	28.8% (334/1159)	13.7% (159/1159)
Clinda	57.1% (356/624)	29.2% (182/624)	13.8% (86/624)
Metro	57.9% (310/535)	28.4% (152/535)	13.6% (73/535)

## Data Availability

This research study was sponsored by Evvy, and the authors of the paper who have access to the data are employees or scientific collaborators of Evvy who have signed contracts with Evvy to be bound by Evvy’s privacy policy and access restrictions. Additional data can be made available through a Data Transfer Agreement that protects the privacy of participants’ data; interested researchers may make requests by contacting kate@evvy.com. The information provided by interested researchers will be used to generate a Data Transfer Agreement (DTA). The DTA protects the privacy of the participants’ data and will need to be signed by both the institution and Evvy before data can be transferred. Additional specifications for laboratory protocols and bioinformatic pipelines can be made available upon request.

## Supplementary Materials

The following supporting information can be downloaded at: https://www.mdpi.com/article/10.3390/microorganisms13071623/s1, Supplementary Figure S1: UMAP analysis including all patients and all timepoints; Supplementary Table S1: Additional demographic data, Supplementary Table S2: Comorbidities, Supplementary Table S3: Statistics for all species presented in Figure 2, Figure 3 and Figure 4, Supplementary Table S4: Pathogenic aerobes and anaerobes, Supplementary Table S5: Specific symptom categories for patient-reported adverse events.

## Abbreviations

The following abbreviations are used in this manuscript:

BV	bacterial vaginosis
VMB	vaginal microbiome
NGS	next-generation sequencing
