# Supplementary material for: Remote BV Management via Metagenomic Vaginal Microbiome Testing and Telemedicine
Source: Microorganisms. 2025 Jul 9;13(7):1623. doi: 10.3390/microorganisms13071623 (PMC12298078; doi:10.3390/microorganisms13071623)
Supplement: Supplementary file 1 [file microorganisms-13-01623-s001.zip › microorganisms-3659165-supplementary/SupplementaryData_Thomas-White_BVManagement/SupplementaryData_Thomas-WhiteBVManagement.pdf]

Supplementary Information

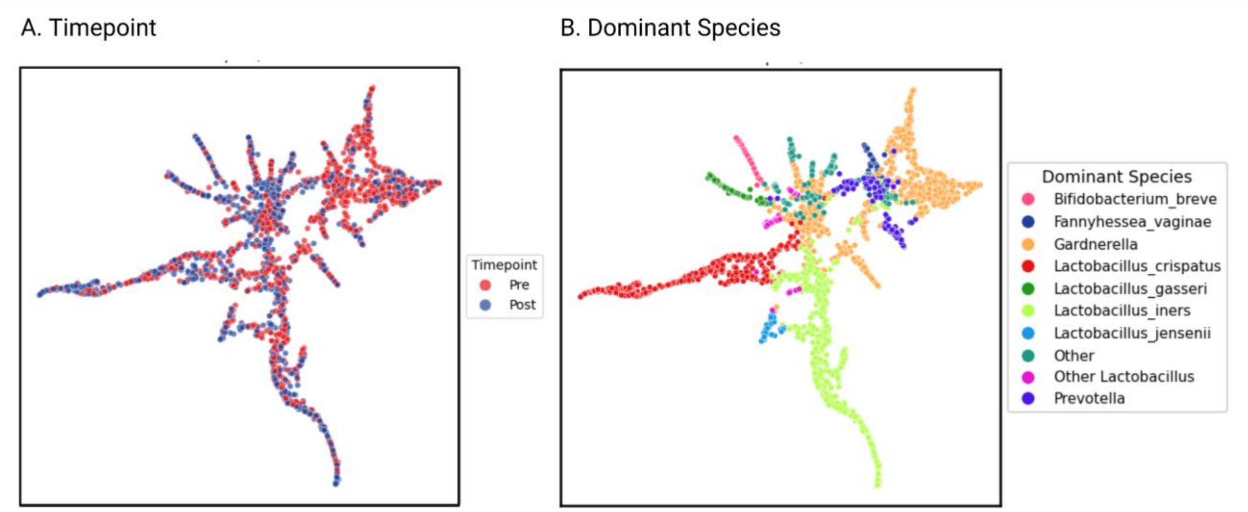

Figure S1. UMAP analysis including all patients and all timepoints. (**a**) Samples color coded based on baseline and followup timepoints. (**b**) Samples color coded based on dominant taxa with dominance was defined as >30% relative abundance.

	**Total** (N=1159)
**Pregnancy Status**	
Actively trying to get pregnant	3.1% (36)
Currently pregnant	0
I am postpartum	2.6% (30)
I am done having children	34.4% (399)
Not interested in pregnancy (now or ever)	21.9% (254)
Not trying to get pregnant (but want to one day)	30.3% (351)
Interested in trying to get pregnant soon (in the next year)	8.7% (101)
I don't know	5.9% (68)
**Sexual Activity**	
Never sexually active	4.3% (50)
Previously sexually active (over a month ago)	26.7% (310)
Within the past 30 days	13.0% (151)
Within the past 2 weeks	20.3% (235)
Within the past 5 days	33.6% (390)
Prefer not to say	2.0% (23)
**Sexual partners**	
Single partner	60.8% (705)
Multiple Partners	5.7% (66)
Other responses*	33.5% (388)
**Any products in the last 30 days**	

Antibiotics	21.7% (252)
Antifungals	17.5% (203)
Boric acid suppositories	39.4% (457)
Feminine hygiene products (wash, spray, wipes, douching, etc.)	15.2% (176)
Hormones (injection, oral, vaginal, topical)	9.3% (108)
None	19.0% (220)
Other	6.0% (69)
Probiotics	57.2% (663)
**Had a period in the last 30 days**	77.7% (900)
Excluding patients reporting menopause	77.4% (897)
**Contraception**	
Any hormonal method	21.7% (252)
No contraceptive method	46.7% (541)
Barrier method	23.3% (270)
Surgical methods (i.e. partner vasectomy, hysterectomy, tubal ligation)	13.7% (159)

*Other response includes those that are not sexually active or preferred not to answer the question

Table S1. Additional Demographics Data

	**Total** (N=1159)
Anxiety	35.9% (416)
Depression	24.5% (284)
Atrophic Vaginitis	1.5% (17)
Vulvodynia	4.5% (52)
Diabetes	0
Breast Cancer	0.8% (9)
Cervical Cancer	0
CIN 1-3	2.5% (29)
Crohn's or IBS	5.5% (64)
Endometrial Cancer	0
Endometriosis/Adenomyosis	6.8% (79)
FHA	0.9% (11)
Genical Herpes	9.6% (111)
HIV	0
Hypothyroidism or Grave's	7.2% (83)
Infertility or Subfertility	4.9% (57)
IC/PBS	4.3% (50)
None	30.2% (350)
Other	6.7% (78)
Ovarian Cancer	0
PID	2.4% (28)
PCOS	7.9% (91)

PMDD	2.8% (32)
SIBO	3.1% (36)
Vaginal Prolapse	1.3% (15)
Vaginismus	2.0% (23)

Table S2. Comorbidities

Table Available as a separate excel file

Table S3. P-values for all species presented in Figures 2-4. These analyses measure the change in relative abundance before and after treatment. Adjusted p-values were determined using a Bonferroni corrected Wilcoxon rank-sum test. These p-values were measured using the full dataset.

Table Available as a separate excel file

Table S4. List of pathogenic aerobes and anaerobes used to make Figure 2.

	Abnormal or increased discharge	Vaginal or vulvar irritation	Vaginal pain, vaginal pressure, or pain with sex	GI Distress	Urinary	Dizziness, lightheadedness, headache	Skin Irritation	Other
**Total**	12.9% (149/1159)	22.9% (265/1159)	5.1% (59/1159)	7.2% (84/1159)	4.7% (54/1159)	1.4% (16/1159)	2.0% (23/1159)	6.2% (72/1159)
**Clinda**	12.7% (79/624)	22.9% (143/624)	5.8% (36/624)	7.9% (49/624)	4.6% (29/624)	1.1% (7/624)	2.1% (13/624)	5.9% (37/624)
**Metro**	13.1% (70/535)	22.8% (122/535)	4.3% (23/535)	6.5% (35/535)	4.7% (25/535)	1.7% (9/535)	1.9% (10/535)	6.5% (35/535)

Table S5: Specific Symptoms of Patient Reported Adverse Events.
