# Supplementary figures and images for: Remote BV Management via Metagenomic Vaginal Microbiome Testing and Telemedicine
Source: Microorganisms. 2025 Jul 9;13(7):1623. doi: 10.3390/microorganisms13071623 (PMC12298078; doi:10.3390/microorganisms13071623)

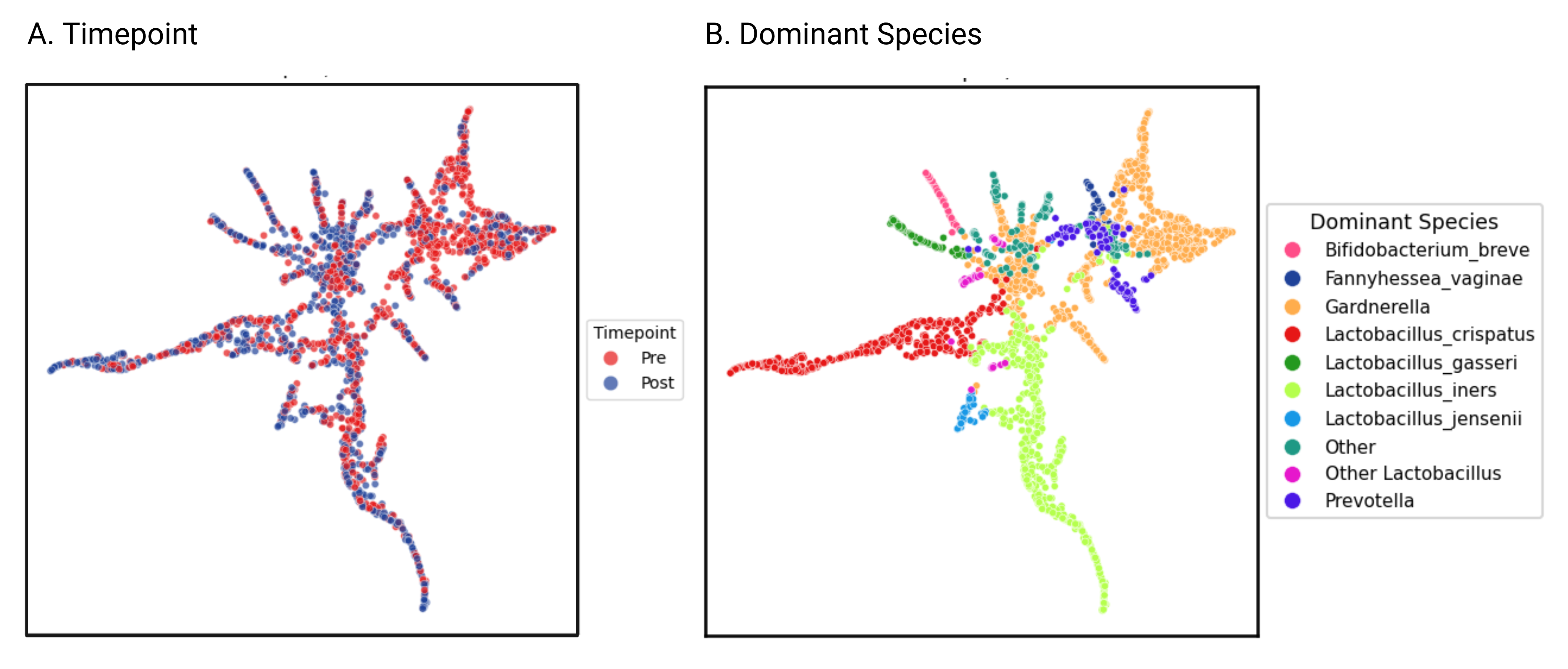

Supplement: Supplementary file 1 [file microorganisms-13-01623-s001.zip › microorganisms-3659165-supplementary/SupplementaryData_Thomas-White_BVManagement/SuppFig1_BVManagement.png]
